# Supplementary material for: 25-Hydroxycholesterol amplifies microglial IL-1β production in an apoE isoform-dependent manner
Source: J Neuroinflammation. 2020 Jun 17;17:192. doi: 10.1186/s12974-020-01869-3 (PMC7298825; doi:10.1186/s12974-020-01869-3)
Supplement: Supplementary file 1 — Additional file 1: Figure S1. a). Expression of CH25H, Cyp27a1 and Cyp7b1 in different cell types in brain based on the Stanford transcriptome database generated by Barres and colleagues (http://www.brainrnaseq.org). b). GC-MS analysis of 25-HC levels in the conditioned medium (left) and total protein levels of cell lysate of primary mouse microglia from wild-type and CH25H-/- mice treated with LPS (0, 0.1, 1, 10, 100, 1000ng/ml). Figure S2a. Differential expression of CH25H or its ortholog in a comparison within one of the datasets. Fold-Changes are relative to non-transgenic, untreated, normal, adult, cortical or parenchymal microglia as appropriate, or, for the last two comparisons, relative to non-myeloid CNS cells (Friedman, et al Cell Report, 2018) http://researchpub.gene.com/BrainMyeloidLandscape. 2b. Relative CH25H gene expression in nonphagocytic (NP) and phagocytic (P) wild-type (WT) or apoe-/- (KO) microglia by RNA seq analysis data generated by Krasemann et al, (Immunity, 47:566, 2017). Figure S3. A schematic diagram for the mechanism associated with 25-HC in amplifying IL-1b production via inflammasome activation. [file 12974_2020_1869_MOESM1_ESM.pdf]

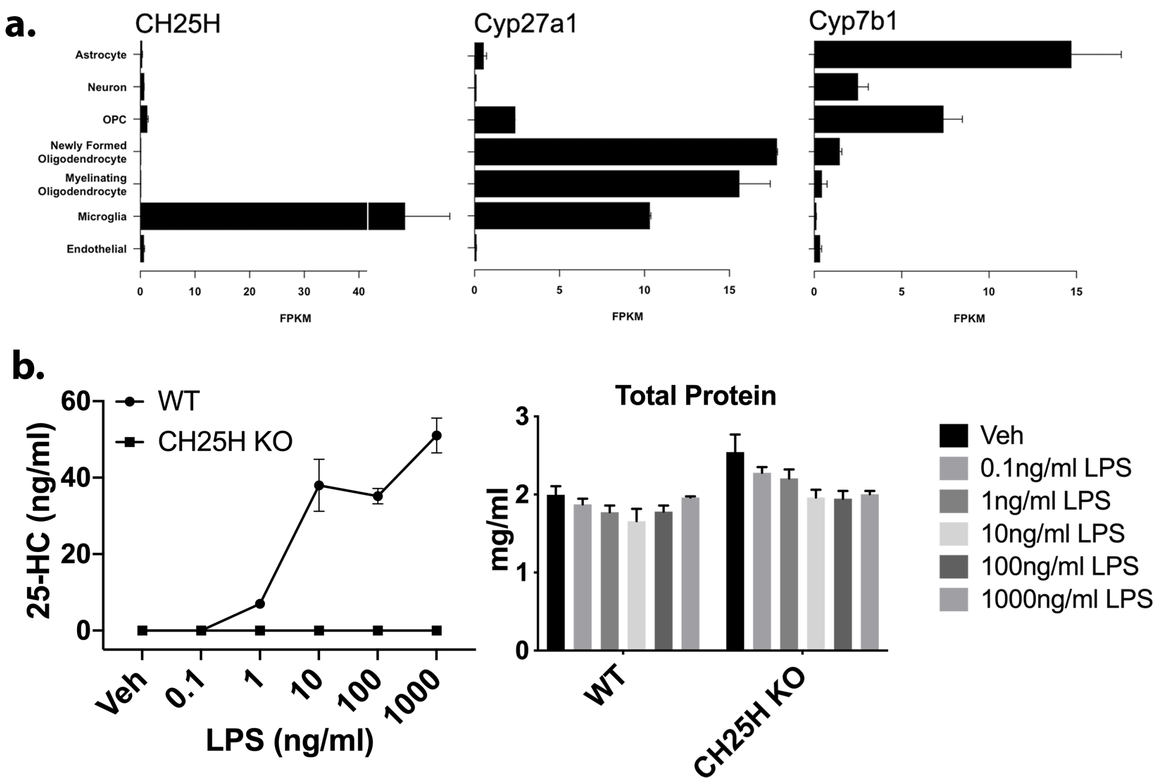

Supplementary Figure 1. a). Expression of CH25H, Cyp27a1 and Cyp7b1 in different cell types in brain based on the Stanford transcriptome database generated by Barres and colleagues (http://www.brainrnaseq.org). b). GC-MS analysis of 25-HC levels in the conditioned medium (left) and total protein levels of cell lysate of primary mouse microglia from wild-type and CH25H-/- mice treated with LPS (0, 0.1, 1, 10, 100, 1000ng/ml).

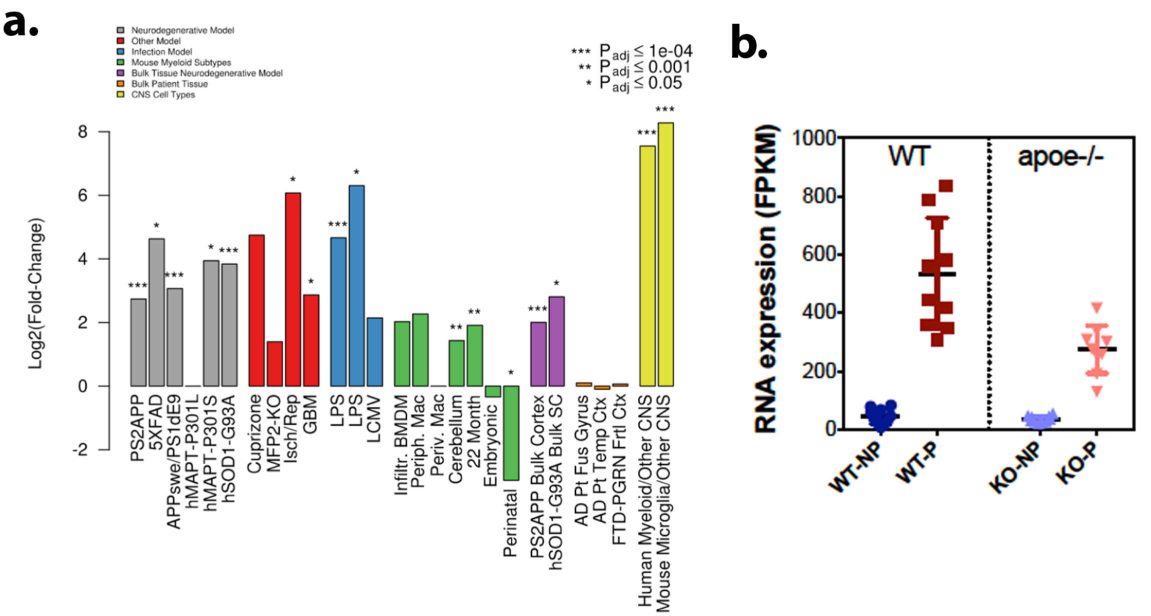

Supplementary Figure 2a. Differential expression of CH25H or its ortholog in a comparison within one of the datasets. Fold-Changes are relative to non-transgenic, untreated, normal, adult, cortical or parenchymal microglia as appropriate, or, for the last two comparisons, relative to non-myeloid CNS cells (Friedman, et al Cell Report, 2018) http://research-pub.gene.com/BrainMyeloidLandscape. **2b.** Relative CH25H gene expression in non-phagocytic (NP) and phagocytic (P) wild-type (WT) or apoe-/- (KO) microglia by RNA seq analysis data generated by Krasemann et al, (Immunity, 47:566, 2017).

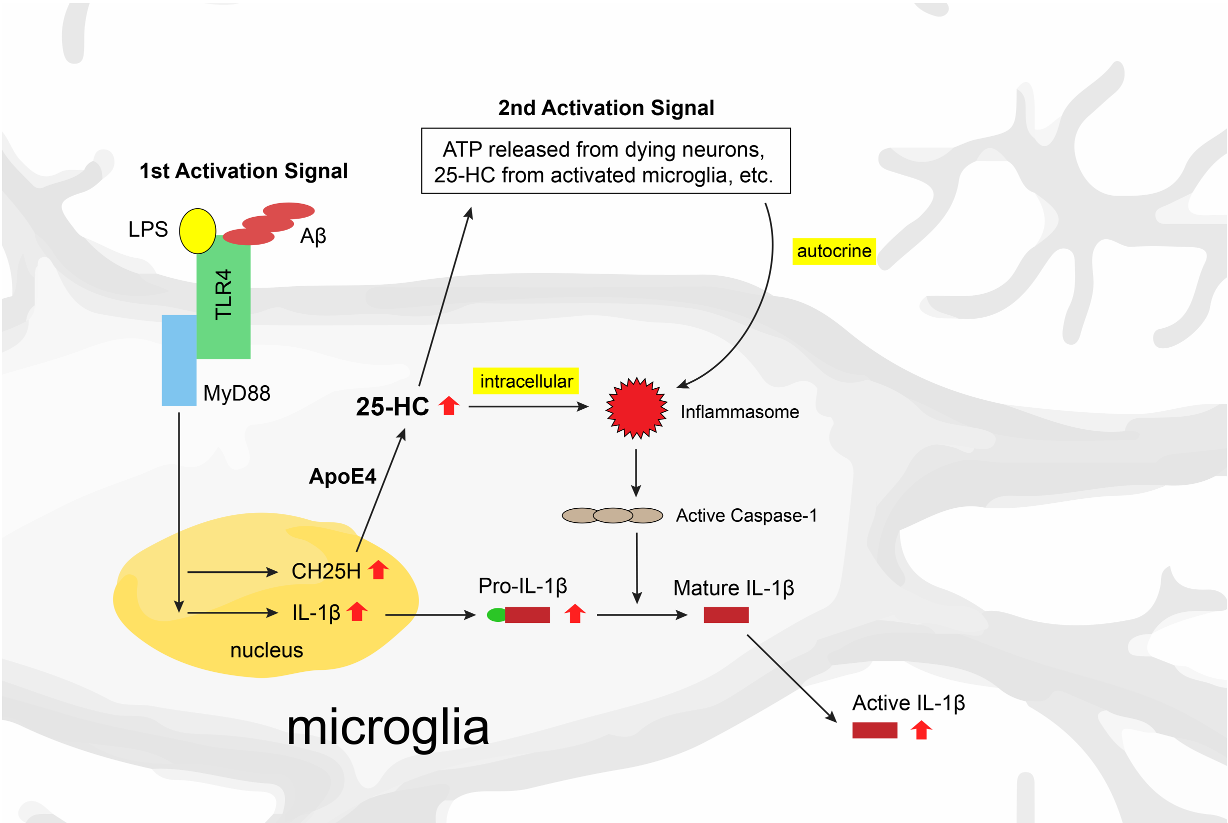

Supplementary Figure 3. A schematic diagram for the mechanism associated with 25-HC in amplifying IL-1β production via inflammasome activation.

.
